# Supplementary material for: Genetic Evaluation of Resilience Indicators in Holstein Cows
Source: Animals (Basel). 2025 Feb 25;15(5):667. doi: 10.3390/ani15050667 (PMC11899513; doi:10.3390/ani15050667)

Table S1: Output Tables and Figures of Proc Univariate for the log-transformed variance (Var, lnvarpred) of daily milk yields .

The UNIVARIATE Procedure
Variable: Var

Moments

**N**	3347	**Sum Weights**	3347
**Mean**	2.40758135	**Sum Observations**	8058.17477
**Std Deviation**	0.81890874	**Variance**	0.67061152
**Skewness**	0.35571539	**Kurtosis**	0.42485699
**Uncorrected SS**	21644.5775	**Corrected SS**	2243.86616
**Coeff Variation**	34.0137516	**Std Error Mean**	0.01415493

Basic Statistical Measures

**Location**		**Variability**	
**Mean**	2.407581	**Std Deviation**	0.81891
**Median**	2.370109	**Variance**	0.67061
**Mode**	.	**Range**	6.14993
		**Interquartile Range**	1.08617

Tests for Location: Mu0=0

**Test**	**Statistic**		**p Value**	
**Student's t**	**t**	170.0879	**Pr > |t|**	<.0001
**Sign**	**M**	1671.5	**Pr >= |M|**	<.0001
**Signed Rank**	**S**	2801424	**Pr >= |S|**	<.0001

Quantiles (Definition 5)

**Level**	**Quantile**
**100% Max**	5.685335
**99%**	4.611152
**95%**	3.830254
**90%**	3.449235
**75% Q3**	2.918899
**50% Median**	2.370109
**25% Q1**	1.832730
**10%**	1.398530
**5%**	1.151754

Quantiles (Definition 5)

Level	Quantile
**1%**	0.659771
**0% Min**	-0.464599

Extreme Observations

Lowest		Highest	
**Value**	**Obs**	**Value**	**Obs**
-0.46459914	1335	5.33941	1924
-0.08228382	791	5.40260	2672
0.00967502	1690	5.47711	1016
0.11110367	3043	5.64507	461
0.19690873	1546	5.68533	1774

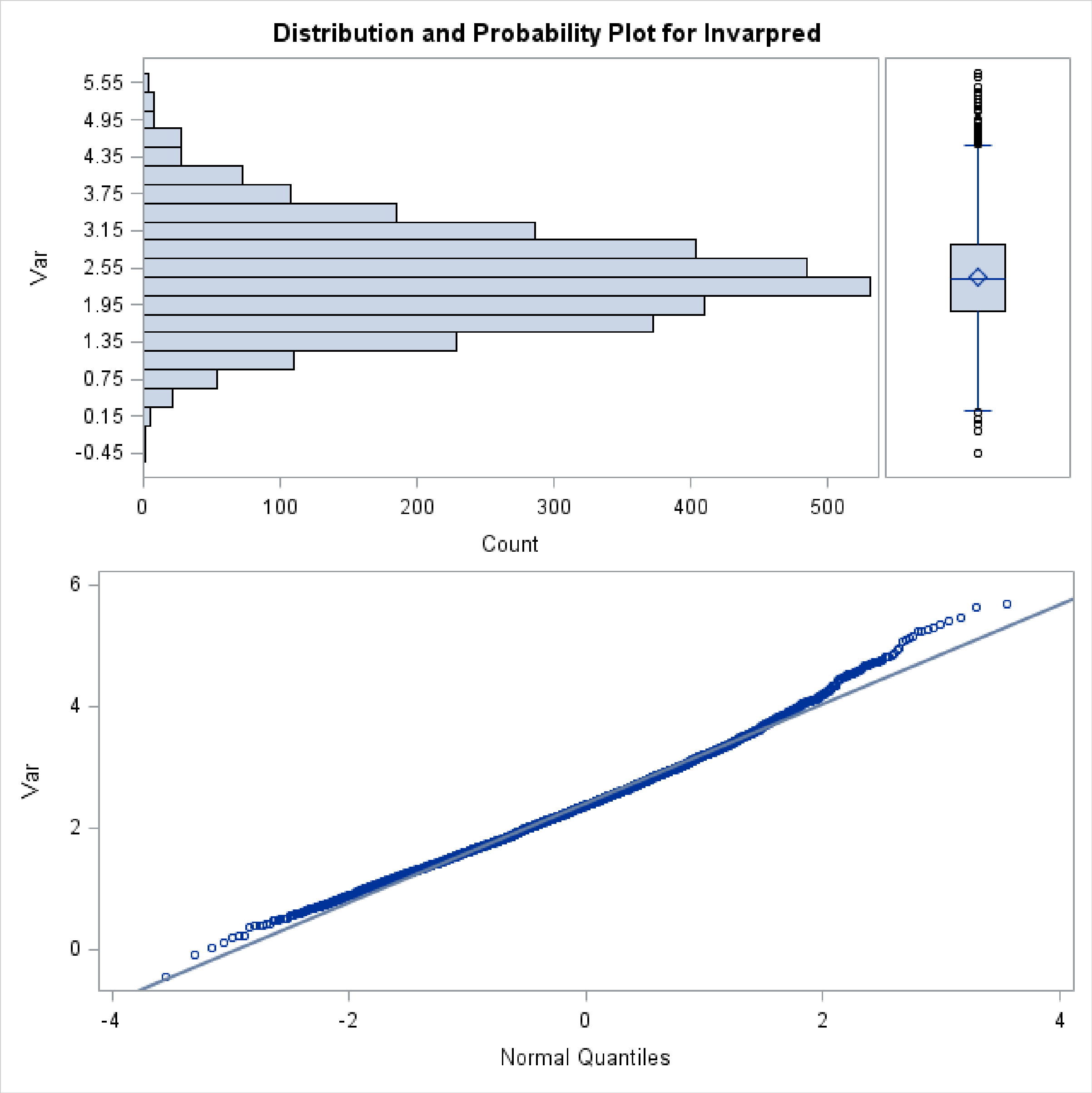
Distribution and Probability Plot for Invarpred
Var
5.55
4.95
4.35
3.75
3.15
2.55
1.95
1.35
0.75
0.15
-0.45
0
100
200
300
400
500
Count
6
4
2
0
-4
-2
0
2
4
Normal Quantiles

The SAS System

The UNIVARIATE Procedure

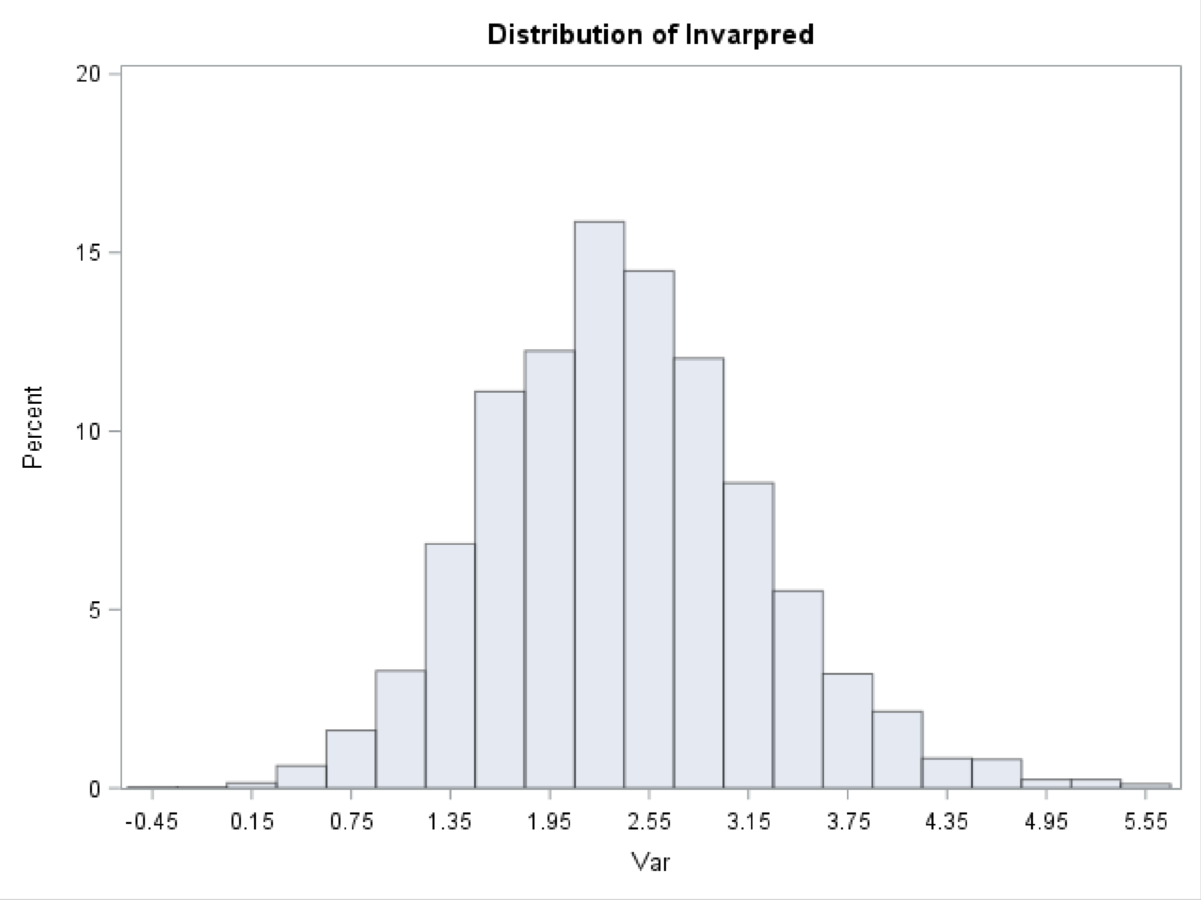

Table S2: Output Tables and Figures of Proc Univariate for the natural log-transformed variance (LnVar, LnVarlg) of deviations from predicted lactation curve.

The UNIVARIATE Procedure
Variable: LnVar

Moments

**N**	3347	**Sum Weights**	3347
**Mean**	1.87823612	**Sum Observations**	6286.45629
**Std Deviation**	0.74410017	**Variance**	0.55368506
**Skewness**	0.30936889	**Kurtosis**	0.52321571
**Uncorrected SS**	13660.0795	**Corrected SS**	1852.63022
**Coeff Variation**	39.6169663	**Std Error Mean**	0.01286185

Basic Statistical Measures

Location		Variability	
**Mean**	1.878236	**Std Deviation**	0.74410
**Median**	1.855517	**Variance**	0.55369
**Mode**	.	**Range**	6.17723
		**Interquartile Range**	0.95955

Tests for Location: Mu0=0

Test		Statistic	p Value	
**Student's t**	**t**	146.0315	**Pr > |t|**	<.0001
**Sign**	**M**	1660.5	**Pr >= |M|**	<.0001
**Signed Rank**	**S**	2800675	**Pr >= |S|**	<.0001

Quantiles (Definition 5)

Level	Quantile
**100% Max**	5.062030
**99%**	3.925442
**95%**	3.124084
**90%**	2.820677
**75% Q3**	2.336118
**50% Median**	1.855517
**25% Q1**	1.376573
**10%**	0.971682
**5%**	0.709723

Quantiles (Definition 5)

Level	Quantile
**1%**	0.281550
**0% Min**	-1.115202

Extreme Observations

Lowest		Highest	
**Value**	**Obs**	**Value**	**Obs**
-1.115202	1335	4.53261	982
-0.478936	791	4.59016	1014
-0.381900	1326	4.94352	1774
-0.343850	1948	5.03723	461
-0.246651	2522	5.06203	456

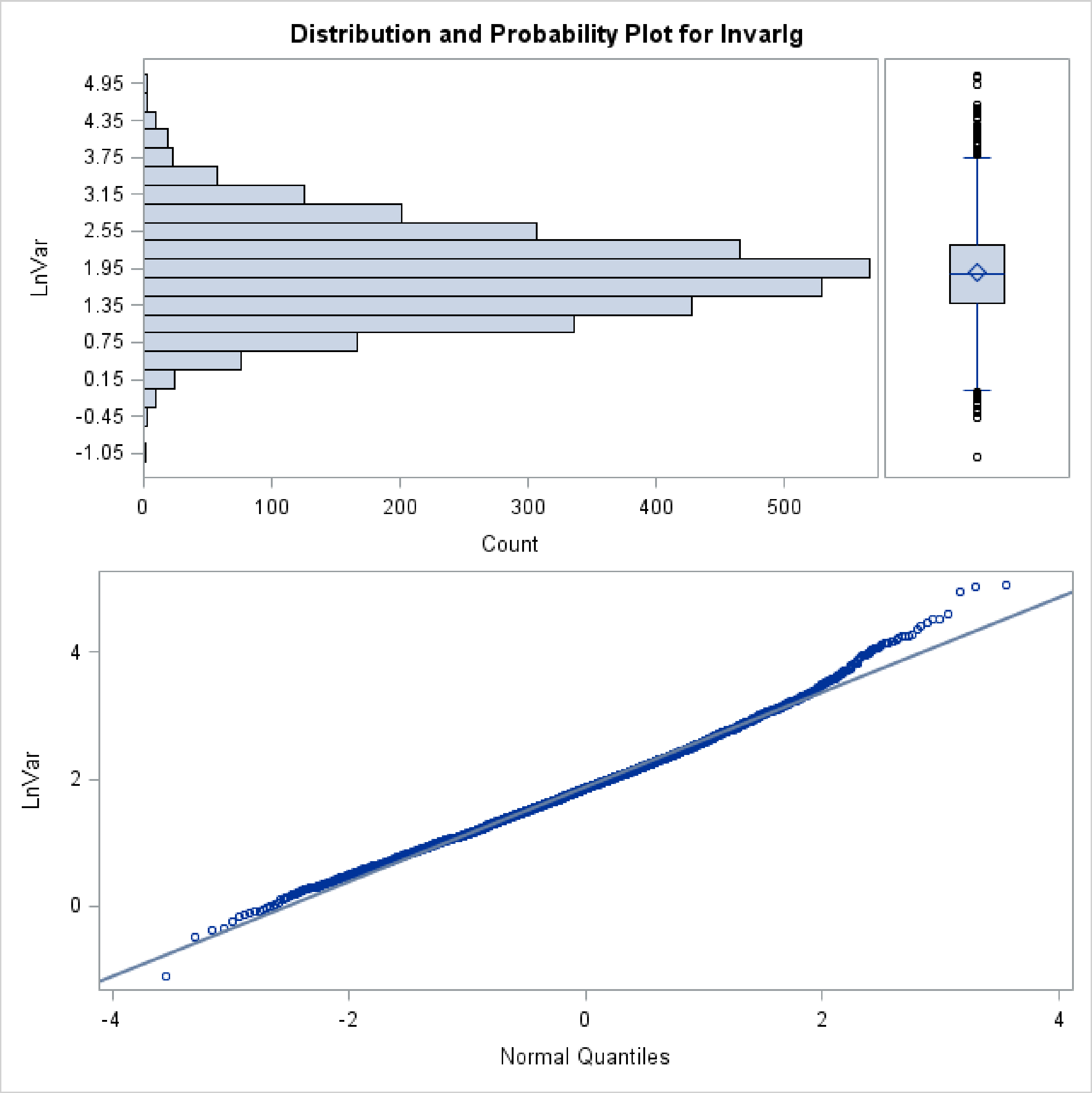
Distribution and Probability Plot for lnvarlg
4.95
4.35
3.75
3.15
2.55
1.95
1.35
0.75
0.15
-0.45
-1.05
LnVar
0
100
200
300
400
500
Count
4
2
0
LnVar
-4
-2
0
2
4
Normal Quantiles

The UNIVARIATE Procedure

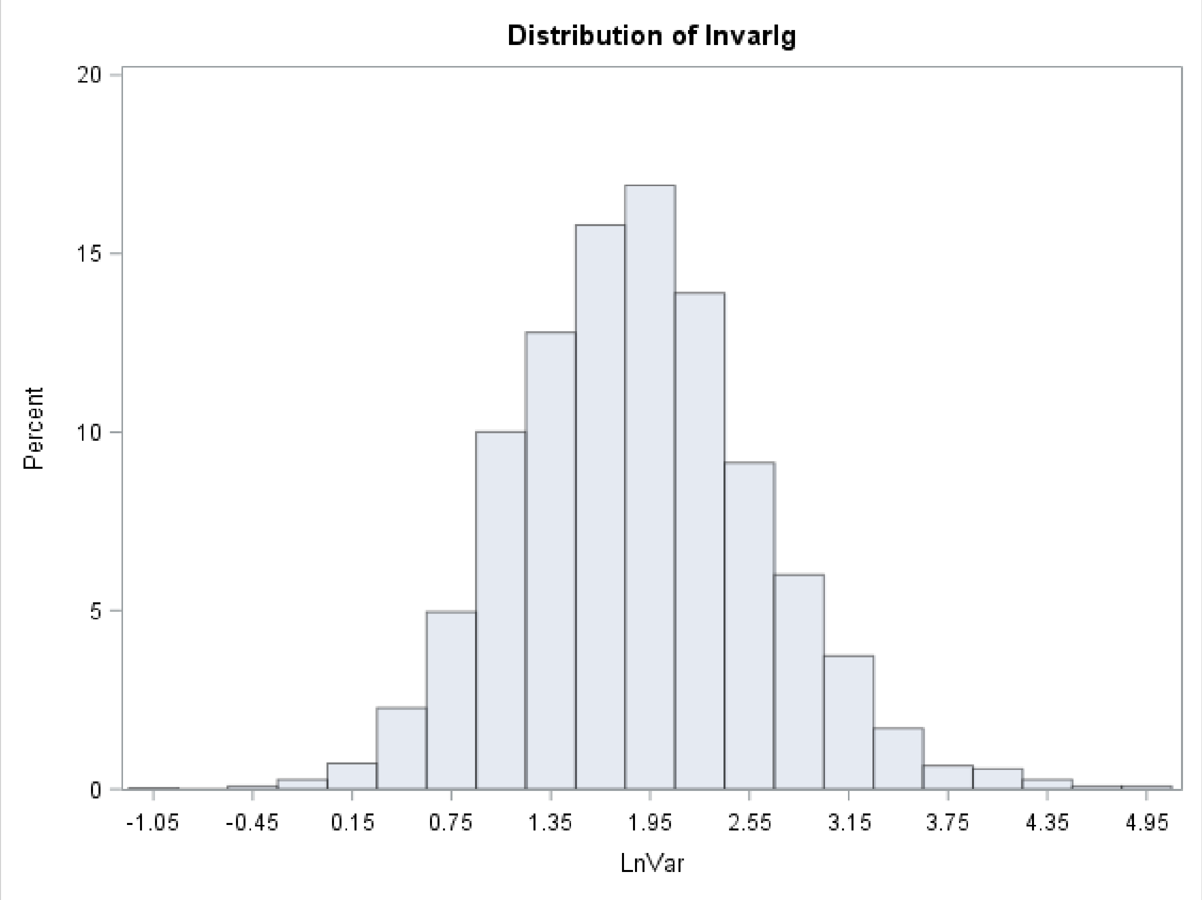

Table S3: Output Tables and Figures of Proc Univariate for the skewness (skew, SKM) of daily milk yields deviations from predicted lactation curve.

The UNIVARIATE Procedure

Variable: Skew

Moments

**N**	3347	**Sum Weights**	3347
**Mean**	-0.8119744	**Sum Observations**	-2717.6784
**Std Deviation**	1.0791808	**Variance**	1.1646312
**Skewness**	0.09933929	**Kurtosis**	3.11479396
**Uncorrected SS**	6103.54138	**Corrected SS**	3896.856
**Coeff Variation**	-132.90822	**Std Error Mean**	0.01865376

Basic Statistical Measures

**Location**		**Variability**	
**Mean**	-0.81197	**Std Deviation**	1.07918
**Median**	-0.71542	**Variance**	1.16463
**Mode**	.	**Range**	11.36197
		**Interquartile Range**	1.10105

Tests for Location: Mu0=0

**Test**	**Statistic**		**p Value**	
**Student's t**	**t**	-43.5287	**Pr > |t|**	<.0001
**Sign**	**M**	-1187.5	**Pr >= |M|**	<.0001
**Signed Rank**	**S**	-2203994	**Pr >= |S|**	<.0001

Quantiles (Definition 5)

**Level**	**Quantile**
**100% Max**	5.628854
**99%**	2.322698
**95%**	0.742868
**90%**	0.188664
**75% Q3**	-0.253611
**50% Median**	-0.715419
**25% Q1**	-1.354665
**10%**	-2.129832
**5%**	-2.640885

Quantiles (Definition 5)

Level	Quantile
**1%**	-3.814686
**0% Min**	-5.733116

Extreme Observations

Lowest		Highest	
**Value**	**Obs**	**Value**	**Obs**
-5.73312	1670	4.14640	1384
-5.37976	663	4.71411	920
-5.12531	1333	4.78277	530
-4.87300	699	4.96073	2416
-4.84035	1967	5.62885	1253

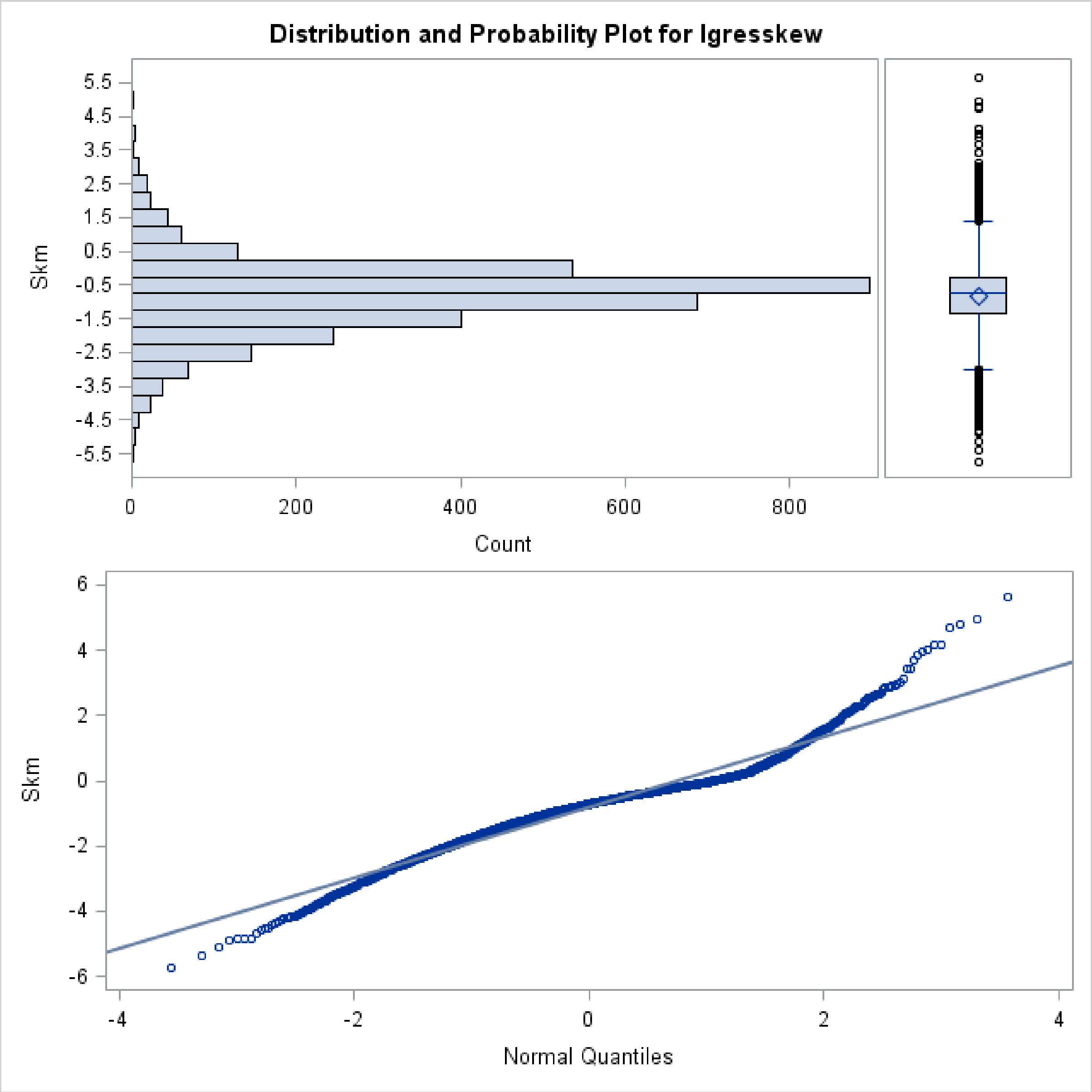

Distribution and Probability Plot for lgresskew
Skm
5.5
4.5
3.5
2.5
1.5
0.5
-0.5
-1.5
-2.5
-3.5
-4.5
-5.5
0
200
400
600
800
Count
6
4
2
0
-2
-4
-6
-4
-2
0
2
4
Normal Quantiles

The UNIVARIATE Procedure

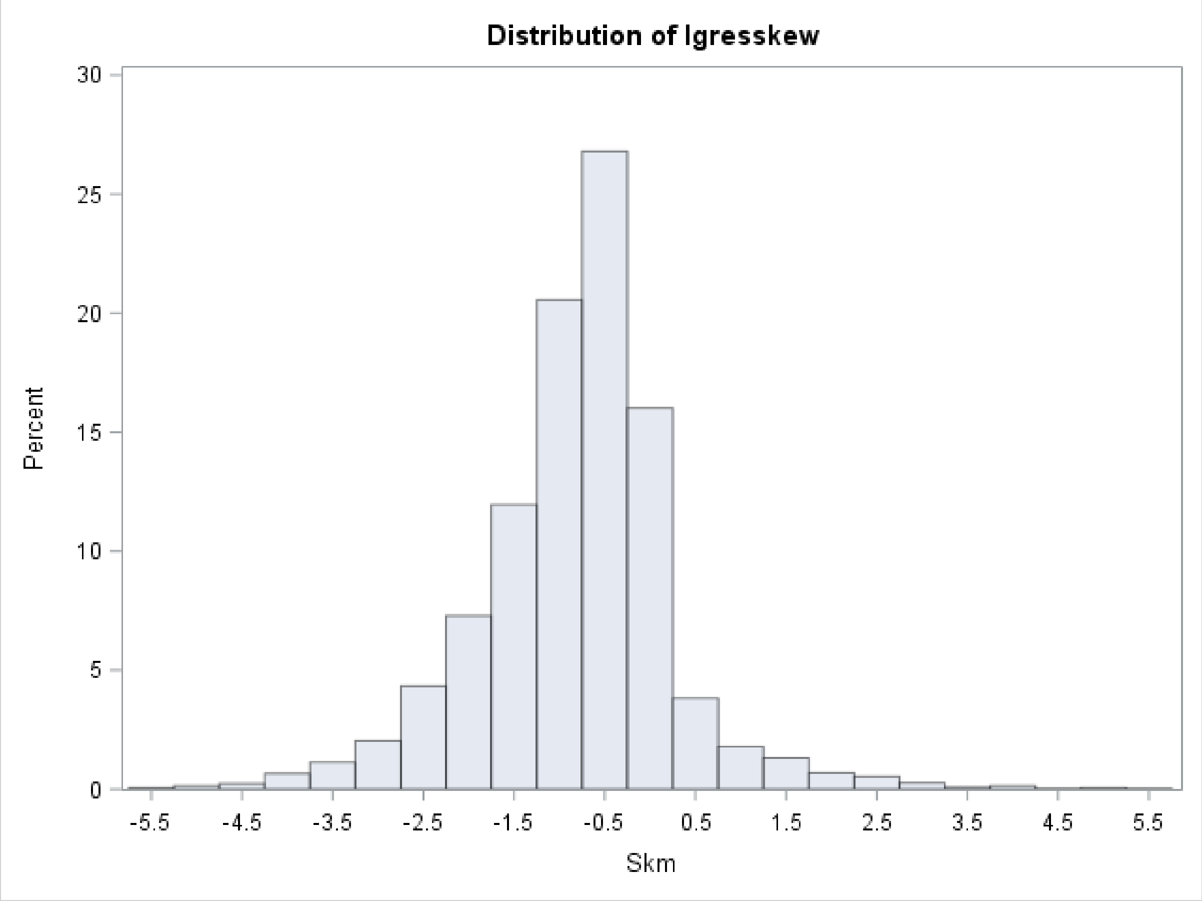

Table S4: Output Tables and Figures of Proc Univariate for the lag-1 autocorrelation (r_auto, lg_auto) of daily milk yields deviations from predicted lactation curve.

The UNIVARIATE Procedure
Variable: r_auto

Moments

**N**	3347	**Sum Weights**	3347
**Mean**	0.30612671	**Sum Observations**	1024.6061
**Std Deviation**	0.22742796	**Variance**	0.05172348
**Skewness**	0.0075447	**Kurtosis**	-0.4049264
**Uncorrected SS**	486.726053	**Corrected SS**	173.066756
**Coeff Variation**	74.2920996	**Std Error Mean**	0.00393112

Basic Statistical Measures

Location		Variability	
**Mean**	0.306127	**Std Deviation**	0.22743
**Median**	0.305283	**Variance**	0.05172
**Mode**	.	**Range**	1.33237
		**Interquartile Range**	0.32823

Tests for Location: Mu0=0

Test	Statistic		p Value	
**Student's t**	**t**	77.8727	**Pr > |t|**	<.0001
**Sign**	**M**	1375.5	**Pr >= |M|**	<.0001
**Signed Rank**	**S**	2650018	**Pr >= |S|**	<.0001

Quantiles (Definition 5)

Level	Quantile
**100% Max**	0.9022536
**99%**	0.8077003
**95%**	0.6830971
**90%**	0.6042089
**75% Q3**	0.4712505
**50% Median**	0.3052828
**25% Q1**	0.1430243
**10%**	0.0117850
**5%**	-0.0614631

Quantiles (Definition 5)

Level	Quantile
**1%**	-0.1923215
**0% Min**	-0.4301155

Extreme Observations

Lowest		Highest	
**Value**	**Obs**	**Value**	**Obs**
-0.430116	26	0.892403	1070
-0.386553	1318	0.893242	3237
-0.357400	2698	0.894521	3207
-0.339914	1346	0.900030	461
-0.319080	2043	0.902254	2272

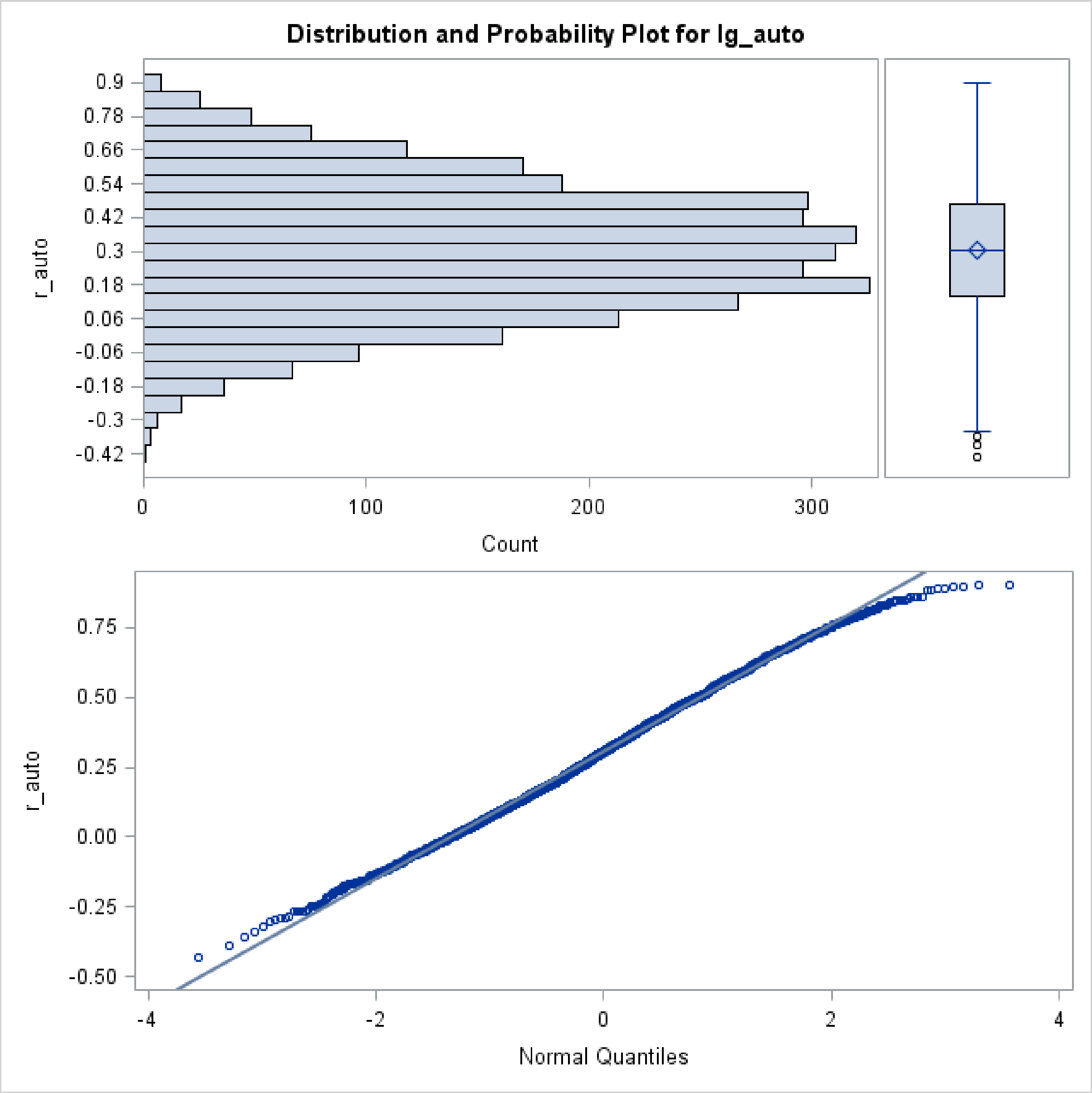
Distribution and Probability Plot for lg_auto
r_auto
0.9
0.78
0.66
0.54
0.42
0.3
0.18
0.06
-0.06
-0.18
-0.3
-0.42
0
100
200
300
Count
r_auto
0.75
0.50
0.25
0.00
-0.25
-0.50
-4
-2
0
2
4
Normal Quantiles

The UNIVARIATE Procedure

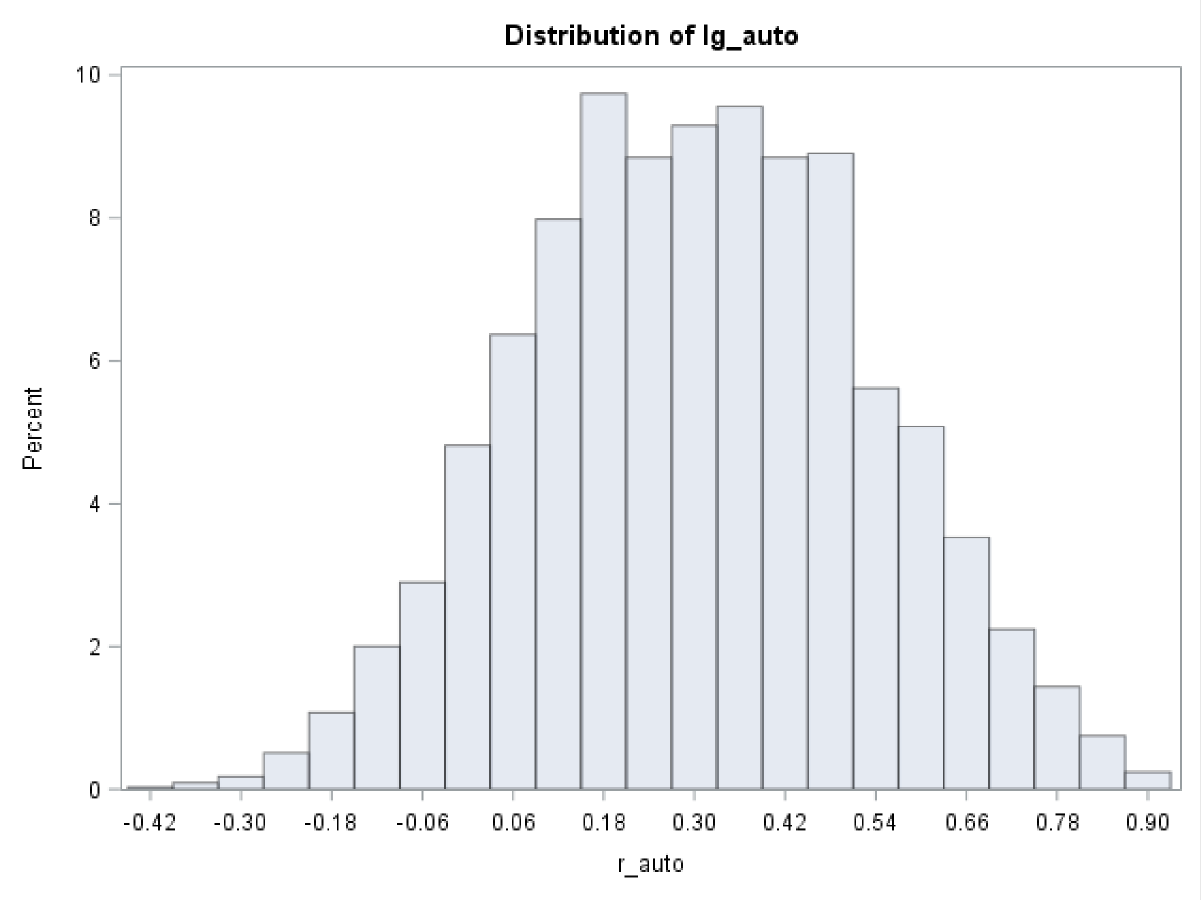

Supplement: Supplementary file 1 [file animals-15-00667-s001.zip › Supplemental S1_res_2025.pdf]
