# Supplementary material for: Genetic Evaluation of Resilience Indicators in Holstein Cows
Source: Animals (Basel). 2025 Feb 25;15(5):667. doi: 10.3390/ani15050667 (PMC11899513; doi:10.3390/ani15050667)
Supplement: Supplementary file 1 [file animals-15-00667-s001.zip › Supplemental S2_res_2025.pdf]

Table S5: Genomic Breeding Value and their accuracy for the log-transformed variance (Var) of daily milk yields.

	Number	Minimum	Maximum	Mean	SD
All animals					
Genomic Breeding Value	31 799	-0,36	0,46	0,02	0,07
Relative Breeding Value	31 799	19,45	169,26	99,83	12,42
Accuracy	22 152	0,02	0,79	0,19	0,12
Sires					
Genomic Breeding Value	6 460	-0,36	0,46	0,02	0,06
Relative Breeding Value	6 460	19,45	169,26	99,83	11,88
Accuracy	5 478	0,02	0,79	0,21	0,10
Cows					
Genomic Breeding Value	25 339	-0,32	0,44	0,02	0,07
Relative Breeding Value Value	25 339	23,61	162,69	99,84	12,55
Accuracy	16 674	0,02	0,49	0,18	0,12
Cows with resilience phenotype					
Genomic Breeding Value	3 080	-0,32	0,44	0,04	0,11
Relative Breeding Value Value	3 080	23,61	162,69	96,59	20,63
Accuracy	3 080	0,13	0,49	0,29	0,09
Genomic Sires					
Genomic Breeding Value	4 629	-0,36	0,38	0,02	0,07
Relative Breeding Value Value	4 629	34,05	169,26	99,37	12,74
Accuracy	4 612	0,02	0,79	0,23	0,09
Genomic Cows					
Genomic Breeding Value	5 216	-0,33	0,42	0,02	0,07
Relative Breeding Value Value	5 216	38,22	152,30	99,39	10,71
Accuracy	5 216	0,02	0,79	0,24	0,09

Table S6: Genomic Breeding Value and their accuracy for the natural log-transformed variance (LnVar) of deviations from predicted lactation curve.

	Number	Minimum	Maximum	Mean	SD
All animals					
Genomic Breeding Value	31 799	-0,35	0,46	0,02	0,07
Relative Breeding Value Value	31 799	31,92	154,63	99,48	10,22
Accuracy	21 321	0,02	0,79	0,19	0,12
Sires					
Genomic Breeding Value	6 460	-0,33	0,46	0,01	0,07
Relative Breeding Value Value	6 460	31,92	152,30	99,77	10,00
Accuracy	5 489	0,02	0,79	0,21	0,10
Cows					
Genomic Breeding Value	25 339	-0,35	0,45	0,02	0,07
Relative Breeding Value Value	25 339	33,27	154,63	99,41	10,27
Accuracy	16 742	0,02	0,49	0,19	0,12
Cows with resilience phenotype					
Genomic Breeding Value	3 080	-0,35	0,45	0,03	0,11
Relative Breeding Value Value	3 080	33,27	154,63	96,80	16,64
Accuracy	3 080	0,14	0,49	0,30	0,09
Genomic Sires					
Genomic Breeding Value	4 629	-0,33	0,42	0,02	0,07
Relative Breeding Value Value	4 629	38,22	152,30	99,39	10,71
Accuracy	4 613	0,02	0,79	0,24	0,09
Genomic Cows					
Genomic Breeding Value	5 216	-0,24	0,45	0,06	0,09
Relative Breeding Value Value	5 216	33,72	137,72	93,49	13,99
Accuracy	5 216	0,10	0,49	0,33	0,06

Table S7: Genomic Breeding Value and their accuracy for.the skewness (skew) of daily milk yields deviations from predicted lactation curve

	Number	Minimum	Maximum	Mean	SD
All animals					
Genomic Breeding Value	31 799	-0,24	0,16	0,00	0,03
Relative Breeding Value Value	31 799	12,46	156,80	99,88	10,72
Accuracy	22 231	0,02	0,80	0,12	0,07
Sires					
Genomic Breeding Value	6 460	-0,24	0,16	0,00	0,03
Relative Breeding Value Value	6 460	12,46	156,80	99,18	11,48
Accuracy	5 200	0,02	0,80	0,12	0,08
Cows					
Genomic Breeding Value	25 339	-0,21	0,15	0,00	0,03
Relative Breeding Value Value	25 339	23,40	154,58	100,06	10,52
Accuracy	17 144	0,02	0,32	0,12	0,07
Cows with resilience phenotype					
Genomic Breeding Value	3 080	-0,21	0,15	0,00	0,04
Relative Breeding Value Value	3 080	23,40	154,58	99,49	15,79
Accuracy	3 080	0,03	0,31	0,14	0,07
Genomic Sires					
Genomic Breeding Value	4 629	-0,14	0,16	0,00	0,03
Relative Breeding Value Value	4 629	47,95	156,80	99,37	12,42
Accuracy	4 309	0,02	0,80	0,13	0,08
Genomic Cows					
Genomic Breeding Value	5 216	-0,21	0,12	-0,01	0,04
Relative Breeding Value Value	5 216	23,40	144,21	97,56	15,58
Accuracy	5 210	0,02	0,32	0,20	0,05

Table S8: Genomic Breeding Value and their accuracy for the lag-1 autocorrelation (r_auto) of daily milk yields deviations from predicted lactation curve

	Number	Minimum	Maximu m	Mean	SD
All animals					
Genomic Breeding Value	31 799	-0,06	0,09	0,00	0,01
Relative Breeding Value Value	31 799	10,67	164,42	100,46	13,26
Accuracy	23 443	0,02	0,76	0,15	0,10
Sires					
Genomic Breeding Value	6 460	-0,06	0,09	0,00	0,01
Relative Breeding Value Value	6 460	10,67	164,42	101,19	13,47
Accuracy	5 562	0,02	0,76	0,17	0,09
Cows					
Genomic Breeding Value	25 339	-0,06	0,09	0,00	0,01
Relative Breeding Value Value	25 339	11,94	159,07	100,27	13,20
Accuracy	17 881	0,02	0,40	0,15	0,10
Cows with resilience phenotype					
Genomic Breeding Value	3 080	-0,06	0,09	0,00	0,02
Relative Breeding Value Value	3 080	11,94	159,07	98,33	20,35
Accuracy	3 080	0,08	0,40	0,23	0,08
Genomic Sires					
Genomic Breeding Value	4 629	-0,06	0,06	0,00	0,01
Relative Breeding Value Value	4 629	37,50	164,42	101,30	14,45
Accuracy	4 586	0,02	0,76	0,19	0,08
Genomic Cows					
Genomic Breeding Value	5 216	-0,06	0,09	0,01	0,02
Relative Breeding Value Value	5 216	11,94	159,07	94,34	17,46
Accuracy	5 216	0,05	0,40	0,27	0,05
